# Microthermoreflectance Characterization of the Band‐Structure Transformations Observed During the Magnetic‐Ordering Transitions of Multilayered 2D Fe_3_GeTe_2_ Ferromagnetic Metals

**DOI:** 10.1002/smsc.202500293

**Published:** 2025-08-13

**Authors:** Ching‐Hwa Ho, Yen‐Chang Su, Yu‐Hung Peng, Zi‐Ying Chen

**Affiliations:** ^1^ Graduate Institute of Applied Science and Technology National Taiwan University of Science and Technology Taipei 106 Taiwan; ^2^ Taiwan Consortium of Emergent Crystalline Materials (TCECM) National Science and Technology Council Taipei 106 Taiwan

**Keywords:** 2D ferromagnetic metals, magnetic properties, optical properties, spin‐polarized band structures, thermoreflectances

## Abstract

2D layered ferromagnetic (FM) materials hold significant promise for various applications owing to their hysteretic behavior and the ultrathin magnetic‐ordering control of spin electrons below the Curie temperature (T_C_). The spin‐polarized order in the FM zone (e.g., T ≤ T_C_ = 225 K for Fe_3_GeTe_2_), paramagnetic (PM) zone (e.g., T > T_C_), and low‐temperature anisotropic (AI) zone (e.g., T < 75 K < T_C_ for Fe_3_GeTe_2_) are typically characterized by observing changes in the magnetization and magnetoresistance under the application of an external magnetic (electric) field. However, observing the near‐band‐edge transitions across the FM, PM, and AI regions of a layered magnetic metal is challenging utilizing optical spectroscopy due to its high surface carrier density for shielding incident lights. In this study, three interband transitions, designated as A1, A2, and A3, are measured and identified through temperature‐dependent microthermoreflectance (μTR) measurements of multilayered Fe_3_GeTe_2_ metal across a temperature range of 20–300 K, enabling the distinction of the FM, PM, and AI zones using optical methods. The A1 transition (≈1.73 eV) is detected across the entire temperature range of 20–300 K, whereas the A2 transition (≈1.95 eV) appears only in the AI zone below 75 K, and the A3 transition (≈2.23 eV) is observed only in the FM phase below T_C_ (≈225 K). Density functional theory calculations suggest that all transitions (A1–A3) originate from the down‐spin‐polarized band of Fe 3*d* electrons. Moreover, temperature‐dependent X‐ray diffraction, Raman, magnetization, and magneto‐resistivity measurements are performed to confirm the T_C_ and AI temperatures observed for multilayered FGT by μTR.

## Graphical abstract

Optical band‐edge transitions of metallic hexagonal Fe_3_GeTe_2_ multilayers are probed by microthermoreflectance spectroscopy across the temperature ranges associated with the paramagnetic, ferromagnetic (FM), and anisotropic (AI) phases. The A1 transition is detected across all three phases, occurring at the Γ point of the FGT band structure. The A2 feature appears only in the AI phase, and A3 is measured only in the AI and FM phases. All three transitions (A1–A3) originate from the spin‐down band structure of Fe_3_GeTe_2_ with Fe *d*‐*d* transitions.

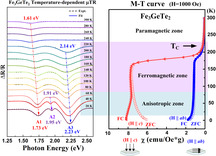

## 1 Introduction

The discovery of graphene^[^ ^1^, ^2^ ^]^ has significantly advanced the research and application of 2D materials,^[^ ^3^, ^4^, ^5^ ^]^ mainly driven by its markedly high electrical conductivity and mechanical strength.^[^ ^6^ ^]^ However, the lack of bandgap limits its application in semiconductor devices and photodetection applications, and the absence of stronger magnetic properties limits its use in memory‐related technologies. Therefore, the development of “2D materials beyond graphene”^[^ ^7^ ^]^ has provided new opportunities for 2D semiconductors and devices,^[^ ^8^, ^9^ ^]^ 2D magnetism (including both ferromagnetic (FM) and antiferromagnetic phases),^[^ ^10^, ^11^ ^]^ 2D layered dichroism with optical‐axis dependence,^[^ ^12^, ^13^, ^14^ ^]^ 2D optical‐excitonic‐series emitters,^[^ ^15^, ^16^ ^]^ visible displays,^[^ ^17^, ^18^ ^]^ near‐infrared light sources,^[^ ^19^ ^]^ and sunlight photocatalysts.^[^ ^20^ ^]^ Among these advanced 2D materials, layered magnetic materials have received special attention owing to their ultrathin, flexible structure and temperature‐dependent magnetic phase, making them highly adaptable to various applications. Specifically, the magnetic polarity can be precisely tuned through external magnetic fields, enabling a wide range of functions in spintronic devices,^[^ ^21^ ^]^ magnetic digital storage units,^[^ ^22^ ^]^ and magnetism quantum materials.^[^ ^23^ ^]^

The 2D magnetic material Fe_3_GeTe_2_ (FGT)^[^ ^24^ ^]^ is one of the three most commonly studied ferromagnetism materials in recent years. Notably, FGT exhibits metallic behavior, distinguishing it from the other two layered magnetic materials, namely CrI_3_ and Cr_2_Ge_2_Te_6_, which are insulators.^[^ ^25^, ^26^ ^]^ FGT was first synthesized and structurally characterized by Deiseroth et al.,^[^ ^27^ ^]^ and its metallic carrier transport was attributed to its high density of active carriers on the basal plane. Thus, it was expected to support high‐efficiency oxygen‐evolution reactions and possess high electrocatalytic activity, even without any chemical treatment.^[^ ^28^ ^]^ Moreover, FGT behaves as a van der Waals (vdW) FM metal, benefiting from its near‐square‐shaped hysteresis loop, large coercivity, and perpendicular magnetic anisotropy,^[^ ^29^ ^]^ making it especially suitable for spintronics devices,^[^ ^30^ ^]^ as well as fundamental magnetic research. In particular, FGT 2D ferromagnets exhibit strong magnetic responses to several environmental stimuli, such as magnetic fields, electric fields, and strain, which makes FGT a favorable candidate for applications in adjustable spintronics, opto‐spintronics, and straintronics.^[^ ^31^, ^32^, ^33^ ^]^ The magnetic order transition temperatures in the Curie temperature (T_C_) and low‐temperature anisotropic (AI) zone may vary and are closely related to the composition and stoichiometry of Fe, Ge, and Te, as found in reported Fe_3‐*x*_GeTe_2_^[^ ^34^ ^]^ and Fe_5‐*x*_GeTe_2_^[^ ^35^ ^]^ through magnetization curves, Raman, and thermoelectric measurements. However, a more powerful technique such as microthermoreflectance (μTR) spectroscopy that can examine the optical band‐structure transitions at different magnetic phases of the multilayer FGT metal with high electron density (i.e., it is not a semiconductor owning an optical gap) would allow accurate, nondestructive, and comprehensive identification of the material's properties before device fabrication.

In this study, high‐quality layered FGT single crystals were grown by chemical vapor transport (CVT) using iodine (I_2_) as the transport agent. Thin nanoflakes of FGT with thicknesses ranging from several to a hundred nanometers were mechanically exfoliated and transferred onto SiO_2_/Si substrates for optical, electrical, and magnetic measurements. The stoichiometry of the FGT was characterized by high‐resolution transmission electron microscopy (HRTEM) combined with energy‐dispersive X‐ray spectroscopy (EDS), and the results closely matched the nominal Fe:Ge:Te ratio of 3:1:2, within a minor standard error. Moreover, temperature‐dependent μTR measurements^[^ ^36^ ^]^ were performed from 20 to 300 K (in steps of 20 K) to evaluate the optical transitions of the low‐temperature AI, FM, and paramagnetic (PM) zones of multilayered FGT in different magnetic phases. In the AI temperature zone below 75 K, three μTR transition features denoted as A1, A2, and A3 were observed between 1.7 and 2.5 eV, whereas only the A1 and A3 transitions were detected in the FM zone of 75–225 K. At 225–300 K, in the PM zone, the A3 feature disappeared and only the A1 feature was distinguished in the μTR spectra. Spin‐polarized band‐structure calculations were conducted for the up‐ and down‐spin electrons to determine the origin of the A1–A3 transitions. The total density‐of‐states (DOS) and projected DOS were also calculated to identify the near‐band‐edge orbital transitions based on the contributions from the three elements in layered FGT. Furthermore, the T_C_ value between the PM and FM zones and the AI transition temperature between the AI and FM zones that obtained by μTR were also verified by temperature‐dependent micro‐Raman (μRaman), temperature‐dependent magnetization, and temperature‐dependent magneto‐resistivity measurements. Overall, the experimental results were consistent with the observed phase‐transition temperatures of optical band‐structure transformation detected by μTR.

## 2 Results and Discussion

**Figure 1:**
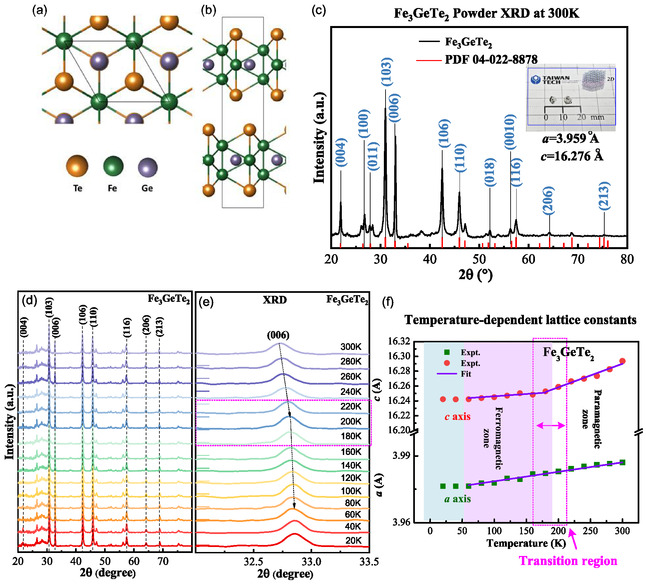
Atomic arrangement and XRD of FGT. a) The top view of hexagonal FGT. The green balls represent Fe atoms, the purple balls represent Ge atoms, and the orange balls represent Te atoms. b) Side view of two‐layer FGT with a vdW gap. c) Powdered XRD pattern of as‐grown FGT with marked peak indices. The red lines show the JCPDS PDF 04‐022‐8878 data, and the inset displays the crystal morphology of layered FGT grown by CVT. d) Temperature‐dependent XRD curves of FGT for 2θ = 20°–80°, measured between 20 and 300 K in steps of 20 K. e) Enlargement of the 32°–33.5° region for the temperature‐angle shift of the (006) peak across the PM and FM zones. f) Calculated lattice constants for the *a*‐ and *c*‐axes estimated from temperature‐dependent XRD in (a). The solid lines represent the linear fits following Vegard's law. The dashed‐line area in (e) and (f) marks the temperature‐transition region between the FM and PM phases of FGT.

**Figure** **1** shows the (a) top view and (b) side view of the atomic arrangement for the crystal structure of FGT, with green, purple, and orange balls depicting the Fe, Ge, and Te atoms, respectively. From the side view, the FGT monolayer can consist of one Fe_3_Ge heterometallic slab sandwiched between two Te layers (orange), and the layers are held together by vdW forces (with vdW gaps) along the *c*‐axis.^[^ ^37^ ^]^ From the top view, the FGT monolayer may contain hexagons composed of its constituent Fe, Ge, and Te ions, forming a standard hexagonal layered structure. The powdered X‐ray diffraction (XRD) pattern of the as‐grown FGT crystal is shown in Figure 1(c), along with the reference data (PDF 04‐022‐8878, (marked by red lines) for comparison. The inset shows the crystal morphology of as‐grown FGT crystals, depicting its shiny surface and layered crystal structure. The lattice constants analyzed from the XRD curve using the hexagonal formula of 
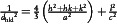
 are determined to be *a *= *b *= (3.959 ± 0.005) Å and *c *= (16.276 ± 0.020) Å, where the *d*_hkl_ is interplanar spacing of each XRD peak indexed in Figure 1(c). The value of the in‐plane lattice constant *a* agrees well with the HRTEM result and matches with the (100) spot observed in the selection‐area electron diffraction (SAED) pattern, as presented in Figure S1 in the Supporting Information. The relatively clear atomic HRTEM image and the dotted spots in the SAED pattern also confirm the high crystallinity of the as‐grown FGT crystals. Notably, the high‐angle annular dark‐field scanning transmission electron microscopy results of the FGT nanoflake, combined with EDS mapping, verify the 3:1:2 stoichiometry of the Fe:Ge:Te compound, within minor standard deviation (Figure S1, Supporting Information). Specifically, the measured composition is 49.66%:16.55%:33.79%, and the ideal stoichiometry is 50.00%:16.67%:33.33%.

The renowned characteristic of FGT is its FM property related to Curie temperature T_C_ to render structural change and spin‐ordering alteration between different magnetic phases.^[^ ^38^ ^]^ To observe the transition‐temperature region and corresponding structure variation of layered FGT, temperature‐dependent XRD measurements were conducted. Figure 1(d) shows the XRD curves (2θ = 20°–80°) measured from 300 to 20 K, in steps of 20 K. The overall pattern, peak indices, and peak intensities remain similar over the whole temperature range, exhibiting the same hexagonal structure shown in Figure 1(c), but slight peak shifts are observed owing to lattice dilation with the increasing temperature. Figure 1(e) shows an enlargement of the temperature‐dependent XRD data at 2θ = 32°–33.5°, highlighting the (006) peak. The peak shift exhibits two different slopes (rates) with the change in temperature, and a transition region is observed at 180–225 K (dashed‐line area). This may be caused by a phase change between the FM and PM zones near T_C_. Temperature‐dependent lattice constants of the hexagonal FGT determined based on the temperature‐dependent XRD curves in Figure 1(d) are calculated and depicted in Figure 1(f). The lattice constants of *a* and *c* in the hexagonal FGT system vary linearly with temperature, consistent with Vegard's law. The solid lines represent the linear fit. The *c*‐axis has different slopes in the PM zone and the FM zone, which are separated by a temperature‐transition region around 175–225 K (dashed‐line area). For the *a*‐axis, there is approximately one slope across the PM and FM regions in Figure 1(f). This indicates that the electron spins of the magnetic moments in layered FGT are mainly oriented along the out‐of‐plane direction of the *c*‐axis, resulting in a significant change from an ordered FM phase to a disordered PM phase for lattice constant *c*. However, for the in‐plane lattice constant *a*, the spin‐order change along the in‐plane direction between the FM and PM phases is minor, as displayed in Figure 1(f). The fitted results for the linear changes in the *a*‐ and *c*‐axes are *a *= 3.97 + 4.26 × 10^−5^ T Å (60 ≤ T ≤ 300 K), *c *= 16.24 + 6.01 × 10^−5^ T Å (FM phase, 60 ≤ T ≤ 180 K), and *c *= 16.19 + 3.17 × 10^−4^ T Å (PM phase, 180 ≤ T ≤ 300 K). The slope is larger for the *c*‐axis in the PM phase than that in the FM phase, suggesting that the free spin order in the PM phase contributes to a larger thermal expansion coefficient in the layered FGT ferromagnet due to the increase of degree of freedom for electrons.

**Figure 2:**
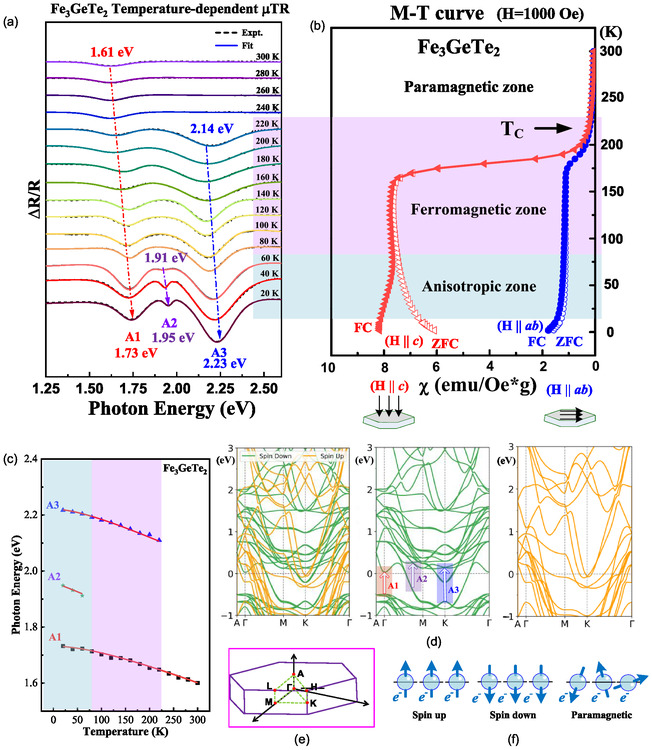
Experimental μTR characterization of multilayered FGT. a) Temperature‐dependent μTR spectra across the AI, FM, and PM phases between 20 and 300 K. The dashed lines represent the experimental data, and the solid curves are fitted to Equation (1). b) In‐plane and out‐of‐plane magnetization curves between 20 and 300 K (M‐T curves) under ZFC and FC conditions. The applied magnetic field strength is H = 1000 Oe, and the measurement configurations (H||*c* and H||*ab*) are depicted in the lower insets. c) Temperature‐dependent transition energies of the A1, A2, and A3 features detected by μTR spectra in (a). The solid lines represent the Varshni fits for the temperature‐energy shifts of the transition energies. The light blue area, light red area, and white area indicate the AI, FM, and PM regions, as found in (b). d) Spin‐polarized band structure of FGT (left). The spin‐down and spin‐up channels are represented in the middle and right figures, respectively. The Fermi level is aligned to 0 eV. e) Brillouin zone of FGT with symmetric points. f) Illustration of the spin‐up, spin‐down, and PM (disordered) electrons in magnetic materials.

To characterize the band‐structure evolution of multilayered FGT during the magnetic‐phase transition, temperature‐dependent μTR measurements,^[^ ^36^ ^]^ and theoretical band‐structure calculations were carried out. **Figure** **2**(a) shows the μTR spectra of a multilayered FGT sample measured between 20 and 300 K, in steps of 20 K. The thickness of the multilayered sample is around 40 nm, estimated by atomic‐force microscopy (AFM), and the optical microscopy (OM) and AFM images are displayed in Figure S2, Supporting Information. Modulation spectroscopy is a powerful tool for the characterization of band‐structure transitions occurring at the critical points of the band structure in solids.^[^ ^39^, ^40^, ^41^ ^]^ The derivative spectral line shape suppresses the unintentional spectral background and emphasizes the direct critical‐point transitions that occur within the band structure.^[^ ^40^ ^]^ In Figure 2(a), the dashed lines represent the experimental μTR spectra obtained at different temperatures, and the solid curves represent the least‐square fits of the experimental data to a derivative Lorentzian line shape function that is appropriate for critical‐point transitions, expressed as follows^[^ ^41^ ^]^

(1) $$\frac{\Delta R}{R}=\text{Re}{\left[\sum_{i=1}^{n}A_{i}^{\text{ex}}e^{-{j\phi }_{i}^{\text{ex}}}(E-E_{i}^{\text{ex}}+{j\Gamma }_{i}^{\text{ex}}\right)}^{-m}]$$

where *m* = 2 implies an excitonic or optical transition for a low‐dimensional layered 2D material, *i* is the respective transition, $A_{i}^{\text{ex}}$ and $\phi_{i}^{\text{ex}}$are the amplitude and phase of the line shape, and $E_{i}^{\text{ex}}$and $\Gamma_{i}^{\text{ex}}$are the energy and broadening parameters of the observed transitions. Three transitions, denoted as A1 = 1.73 eV, A2 = 1.95 eV, and A3 = 2.23 eV, are detected and analyzed in the low‐temperature μTR spectrum at 20 K, as shown in Figure 2(a). With the temperature increase, the A1–A3 features show energy reduction, and the A2 feature is ionized and not detectable at T = 80 K. At T > 220 K, the A3 feature disappears and only one A1 feature is observed in the μTR spectra. The transition energies of A1, A2, and A3 are determined from the fitting using Equation (1) and depicted in Figure 2(c) for illustration. The A2 feature may be detected at 20–60 K because this temperature range is included in the AI zone (light blue region). Moreover, the A3 feature is observed at both 20–60 K (AI) and 80–220 K (FM, light red region). The A1 feature can be detected in all zones of AI, FM, and PM (white region) of multilayered FGT. This means the A1 transition originates from the highest symmetry point (e.g., Γ point) in the layered FGT band structure, which is detected regardless of the change in spin ordering for different magnetic phases.

To verify the temperatures identified for magnetic‐order change in the AI, FM, and PM zones, magnetization measurements with field cooling (FC) and zero‐FC (ZFC) are implemented in the range of 4–300 K (i.e., M‐T curves). The applied magnetic field for the FC condition is H = 1000 Oersted (Oe). Figure 2(b) shows the in‐plane (blue, H||*ab* plane) and out‐of‐plane (red, H||*c*‐axis) M‐T curves under FC and ZFC conditions. The solid symbols represent the M_FC_ data and the open symbols represent the M_ZFC_ data. The lower insets in Figure 2(b) depict the FC schemes for the magnetic field directions of H||c and H||*ab*. As the temperature decreases from 300 K to below T_C_ (≈225 K), the in‐plane and out‐of‐plane magnetic susceptibility (χ)increase owing to the magnetic‐order transition from the PM phase to the FM phase. In particular, the out‐of‐plane susceptibility (red) is much larger than the in‐plane susceptibility (blue) at 4–225 K, verifying the hexagonal 2D layered structure of FGT. This also confirms that the orientations of the magnetic moments for the spin electrons are largely aligned along the out‐of‐plane direction of the *c*‐axis. As shown in Figure 2(b), a higher magnetic susceptibility χ is observed under FC conditions than under ZFC conditions, for both the out‐of‐plane (red color, H||*c*) and in‐plane (blue color) M‐T curves below T_C_. When no magnetic field is applied during cooling (ZFC), electron spins are locked in a slightly disordered state compared with the state of well‐aligned spins under FC conditions. Thus, the χ(FC) values (solid symbols) are larger than the χ(ZFC) values (open symbols), for both the H||c and H||*ab* curves. The small difference between χ(FC) and χ(ZFC) in the AI zone for the in‐plane direction (H||*ab*) implies that most of the magnetic moments are largely aligned along the *c*‐axis of the out‐of‐plane direction. The significant difference between χ(FC) and χ(ZFC) for H||*c* is observed in the AI zone below 75 K. This is indicative of magnetic anisotropy caused by magneto‐crystalline anisotropy in layered FGT.^[^ ^42^ ^]^ The susceptibility difference between χ(FC) and χ(ZFC) at each temperature corresponds to the temperature‐dependent coercivity of FGT in the AI and FM zones. The maximum coercivity occurs at the lowest temperature in the out‐of‐plane direction, as shown in Figure 2(b). From the M‐T curves, the T_C_ between the PM and FM phases occurs around 225 K, and the temperature at the boundary between the AI and FM phases is ≈75 K. These results match well with the transition temperatures obtained from the temperature‐dependent μTR measurements in Figure 2(a).

Figure S3(a) and S3(b), Supporting Information display the magnetization of layered FGT versus the magnetic field strength (M‐H curve) applied along the H||*ab* and H||*c* directions at several temperatures between 10 and 300 K. Figure S3(c), Supporting Information compares the magnetic‐transition behaviors for the H||*ab* and H||*c* conditions in the AI zone at 10 K, and the inset shows the result at 300 K in the PM zone. The H||*c* condition presents a much sharper magnetization transition from negative to positive near H = 0 Oe, indicating the strong magnetic anisotropy of the electron spins in the FM and AI zones of layered FGT. According to Figure S3(a) and S3(b), Supporting Information, a stepping‐like magnetization transition is observed from 10 to 200 K for both the H||*c* and H||*ab* conditions, whereas the M‐H curves exhibit linear behavior between 250 and 300 K, consistent with the PM phase. Thus, the Curie temperature is estimated to be ≈225 K, which agrees well with the experimental observations displayed in Figure 2(a) and (b). Figure S3(d), Supporting Information shows the 1/M versus temperature results derived from Figure 2(b) for the H||*ab* and H||*c* directions. The light red region highlights the transition temperatures between the PM and FM phases around 175–225 K, similar to the μTR result of the band‐structure transition A3, as seen in Figure 2(a). Figure 2(c) depicts the transition energies of A1, A2, and A3 at various temperatures, obtained from the μTR spectra in Figure 2(a) using Equation (1). The solid lines represent the least‐square fits of the experimental data using the Varshni relationship, E_i_(T) = E_i_(0)‐a_i_·T^2^/(b_i_ + T),^[^ ^43^ ^]^ where i signifies A1, A2, or A3, E_i_(0) is the transition energy at 0 K, a_i_ denotes the strength of the electron (exciton)‐phonon interaction, and b_i_ is closely related to the Debye temperature.^[^ ^43^ ^]^ The obtained fitting parameters of the A1, A2 and A3 transitions in Figure 2(c) are E_i_(0) = 1.74, 1.96, and 2.24 eV, *a*_i_ = 7.4 × 10^−4^, 7.4 × 10^−4^, and 7.9 × 10^−4^ eV K^−1^, and *b*_i_ = 200, 195, and 198 K, respectively. The values of a_i_ and b_i_ for the three transitions are comparable to show a parallel temperature‐energy shift in A1, A2, and A3. Therefore, the three transitions may come from the same spin direction of electronic states (e.g., spin‐down electrons).

To characterize the origin of the transition in A1–A3 μTR features that occurred in the band structure of FGT, spin‐polarized band‐structure calculations with up‐ and down‐spins are performed along with the corresponding spin‐polarized DOS computations. Specifically, density functional theory (DFT) calculations are employed to calculate and optimize the geometries and associated energies using Quantum Espresso simulation software.^[^ ^44^ ^]^ Figure 2(d) shows three calculated band structures, namely spin‐down and spin‐up (left), spin‐down‐only (middle), and spin‐up‐only (right), with green lines depicting the spin‐down states and orange lines depicting the spin‐up states. The band structures are calculated along the high symmetry points in the Brillouin zone, as depicted in Figure 2(e). An illustration of spin‐up, spin‐down, and disordered (PM phase) electron states is shown in Figure 2(f). The spin‐up and spin‐down band structures exhibit distinct E‐*k* curves in the calculation results, and this spin splitting of the electronic states may be indicative of the magnetic behavior. From the spin‐polarized band structures of FGT depicted in Figure 2(d), the energy of E = 0 eV for the Fermi level indicates that the conduction band minimum (CBM) is fully occupied with electronic states, inducing the metallic behavior of the layered magnet. Nevertheless, the temperature‐dependent μTR measurement in Figure 2(a) displays of A1, A2, and A3 optical transitions for the AI, FM, and PM zones near the band edge. The A1 feature originates from the band‐to‐band transition that occurs at the Γ point with high symmetry in the down‐spin band of Figure 2(d). The A2 feature is only detected in the AI zone below 80 K, potentially derived from the critical‐point transition of parallel band slopes at the down‐spin band of the Γ‐M direction shown in the middle of Figure 2(d). The A3 feature is observed in the FM and AI zones below 225 K (T_C_). A higher‐energy band‐to‐band transition occurs at the K point, where the E‐*k* curve of the conduction band (CB) state becomes flatter to widen the A3 line feature and weaken its μTR intensity in the PM phase with T > T_C_. Note that the calculated transition energies of A1–A3 in Figure 2(d) are lower than those of the experimental μTR values in Figure 2(a). It is a general issue for DFT calculations, however, the highest DOS peaks for the band edge still provide the highest transition probability for the occurrence of optical transitions A1–A3 during the magnetic‐phase change of the FGT layered magnet. All the A1, A2, and A3 transitions are inferred to occur in the down‐spin band in the middle of Figure 2(d), and total and partial DOS calculations are implemented to verify and identify the transition assignments.

**Figure 3:**
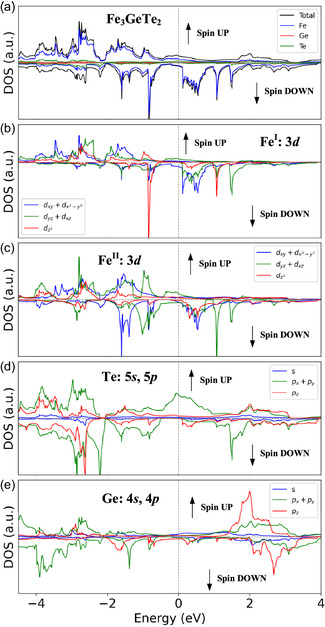
Calculated total and partial DOS of FGT with spin‐up and spin‐down configurations. a) DOS of FGT, Fe, Ge, and Te near the VB and CB band edges. DOS of b) Fe^I^:3*d* and c) Fe^II^:3*d* with electron symmetry in $d_{xy}+d_{x^{2}-y^{2}},d_{yz}+d_{xz}$, and $d_{z^{2}}$. The spin‐up and spin‐down DOS of the d) Te 5*s*, 5*p* with *s*, *p*_x_ + *p*_y_, and *p*_z_ states and e) Ge 4*s*, 4*p* with *s*, *p*_x_ + *p*_y_, and *p*_z_ states.

For matched valence in the FGT compound, the valences of each element can be distinguished as Fe_3_GeTe_2_ = (Fe^2+^)(Fe^3+^)_2_(Ge^4−^)(Te^2−^)_2_, providing the representative scheme of the atomic positions, with two Fe^I^ (Fe^3+^), one Fe^II^ (Fe^2+^), one Ge (Ge^4−^), and two Te (Te^2−^), as depicted in the side view in Figure S4(f), Supporting Information. The X‐ray photoelectron spectroscopy (XPS) results for the (a) full spectrum, (b) Fe 2*p*, (c) Ge 3*d*, (d) Te 3*d*, and (e) valence band (VB) of FGT are shown in Figure S4, Supporting Information for clarification. Essentially, the VB consists of Fe 3*d* and Te 5*p* hybrid states positioned at 0 to ≈10 eV, as depicted in the XPS spectrum in Figure S4(e), Supporting Information. The hybridization of electron orbitals in the VB and CB can be observed and verified by total and partial DOS calculations with up‐ and down‐spin polarizations, as shown in **Figure** **3**. The total and partial DOS were calculated using a dense k‐mesh (16 × 16 × 4), employing the tetrahedron method to sample the Brillouin zone. The calculated spin‐up and spin‐down total and partial DOS of (a) FGT, (b) Fe^I^: 3*d* (with $d_{xy}+d_{x^{2}-y^{2}},d_{yz}+d_{xz}$, and $d_{z^{2}}$), (c) Fe^II^: 3*d* (with $d_{xy}+d_{x^{2}-y^{2}},d_{yz}+d_{xz}$, and $d_{z^{2}}$), (d) Te: 5*s*, 5*p* (with *s*, *p*_*x*_ + *p*_*y*_ and *p*_*z*_), and (e) Ge 4*s*, 4*p* (with *s*, *p*_*x*_ + *p*_*y*_ and *p*_*z*_) are shown in Figure 3. The valence states, spanning from −4.5 to −1.0 eV, are predominantly contributed by the hybridization of Fe 3*d*, Ge 4*p*, and Te 5*p* orbitals. The VB and CB states near the Fermi level (E = 0 eV, marked by a dashed line) are mainly dominated by Fe 3*d* orbitals, with a minor contribution from Te 5*p* orbitals. As shown in Figure 3(a)–(e), the spin‐down orbitals near the VB and CB states for total and partial DOS exhibit an energy gap between two DOS maxima, similar to the bandgap between the CBM and VB maximum (VBM) for a semiconductor DOS. This may help explain why all the observed μTR transitions of the A1, A2, and A3 features occur in the spin‐down band structure with the highest DOS at the VBM, as shown in the middle part of Figure 2(d). Moreover, the Fe^I^:3*d* and Fe^II^:3*d* states at the VBM and CBM are the highest (i.e., separated by an energy gap) for all the DOS in Figure 3. This result confirms that the A1, A2, and A3 transitions originate from Fe *d*–*d* transitions. The Fe *d* electrons may change their spin status during different magnetic phases (AI, FM, and PM), resulting in the disappearance of the A2 and A3 features at 75 and 225 K, as observed in the temperature‐dependent μTR spectra of Figure 2(a). In Figure 3(b) and (c), a significant difference in DOS contributions is observed between Fe^I^:3*d* and Fe^II^:3*d* electrons. The Fe^I^ $d_{z^{2}}$ orbital exhibits a sharp peak around −1 eV, whereas the $d_{z^{2}}$ orbital of Fe^II^ displays a broader distribution across the energy range. This behavior can be attributed to the hybridization of Fe^II^ with the nearest Te atoms, leading to saturation of the $d_{z^{2}}$ orbitals. In contrast, Fe^I^ lacks ligand atoms in the *z*‐direction, yielding a distinct and sharp $d_{z^{2}}$ orbital configuration in Figure 3(b). As shown in Figure S4(f), Supporting Information, Fe^I^ (Fe^3+^) and Fe^II^ (Fe^2+^) may contribute different valences to layered FGT. Thus, the $d_{z^{2}}$ orbital of the Fe^I^ (Fe^3+^) ion may closely affect the spin states, magnetic ordering, and optical transitions of different magnetic phases in the layered FGT ferromagnet.

**Figure 4:**
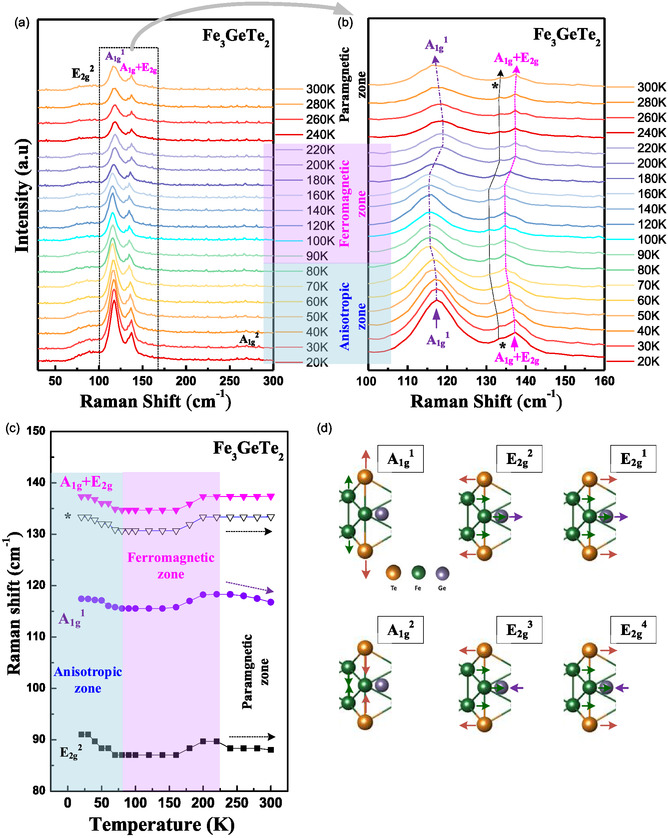
Temperature‐dependent μRaman spectroscopy of multilayered FGT. a) Temperature‐dependent μRaman spectra between 20 and 300 K. b) Enlargement of the 100–160 cm^−1^ region in (a), to observe the abnormal temperature‐frequency shift of the A_1g_^1^, *, and A_1g_ + E_2g_ peaks. c) Temperature‐dependent Raman shift of the Raman peaks detected in (a). All modes exhibit S‐shape temperature‐energy shifts from 20 to 300 K across the AI, FM, and PM regions. d) Illustration of atomic movements for the in‐plane and out‐of‐plane modes in FGT.

Overall, the temperature‐dependent XRD, magnetization, and temperature‐dependent μTR measurements reveal that the boundary temperature between the PM and FM phases is ≈225 K (i.e., T_C_) and that the boundary temperature between the AI and FM phases is ≈75 K, but additional temperature‐dependent μRaman and temperature‐dependent magnetoresistance experiments are implemented to verify these findings and identify the material properties. **Figure** **4**(a) shows the temperature‐dependent μRaman spectra of multilayered FGT, obtained between 20 and 300 K, in the wavenumber range of 40–300 cm^−1^. Three vibration modes are detected at 300 K: E_2g_^2^ at ≈88 cm^−1^, A_1g_^1^ at ≈118 cm^−1^, and A_1g_ + E_2g_ at ≈136 cm^−1^.^[^ ^35^, ^45^ ^]^ Where the standard error for the estimate of Raman wavenumber is ≈ ± 1.0 cm^−1^. The modes shift with the changes in temperature, and the temperature‐energy shift traces of the A_1g_^1^‐ and A_1g_ + E_2g_‐related modes are magnified for the wavenumber range of 100–160 cm^−1^ and displayed in Figure 4(b). There are three vibration peaks of A_1g_^1^, *, and A_1g_ + E_2g_ that can be observed in the temperature‐dependent Raman spectra in Figure 4(b), at different temperature ranges: AI (light blue), FM (light red), and PM (white) zones. Notably, the three Raman peaks reveal S‐shape traces as the temperature increases from 20 to 300 K. All the temperature‐dependent Raman peak shifts of the vibration modes are depicted in Figure 4(c) for comparison. Each of the Raman modes of A_1g_ + E_2g_, *, A_1g_^1^, and E_2g_^2^ reveals an S‐shaped trend as the temperature increases from 20 to 300 K. All the modes cross the AI, FM, and PM zones, with changes in the magnetic ordering. The boundary temperature is ≈75 K between the AI and FM phases and ≈225 K between the FM and PM phases. All the atomic movements of the Raman modes are depicted in Figure 4(d) for comparison. Essentially, the A_g_‐related modes (A_1g_^1^ and A_1g_^2^) display out‐of‐plane atomic movements, whereas the E_g_‐related modes (E_2g_^1^, E_2g_^2^, E_2g_^3^, and E_2g_^4^) exhibit in‐plane atomic vibrations. Figure S5(a), Supporting Information shows the angular‐dependent polarized Raman spectra of FGT from 0° or 180° (E || *a*‐axis) to 90° (E ⊥ *a*‐axis), to clarify the polarized rejection ratio of the A_g_‐ and E_g_‐related modes. The analyzed polar plots of the modes are shown in Figure S5(c)–S5(e), Supporting Information. The A_1g_^1^ mode exhibits the highest polarized rejection ratio compared with those of the E_g_‐related modes owing to its out‐of‐plane characteristic, similar to other 2D materials such as SnS_2_ and SnSe_2_.^[^ ^46^ ^]^ The unusual S‐shape change in the Raman modes with increasing temperature [see Figure 4(b) and (c)] may be attributed to the spin‐order (spin‐moment)‐induced vibration‐frequency change for the different magnetic phases (AI, FM, and PM) of FGT. The temperature‐dependent μTR and Raman results verified that the boundary temperatures for the AI and FM phases and for the FM and PM phases are respectively occurred at 75 and 225 K for the layered FGT. Notably, the enhancement of magnetic anisotropy of electron spins along the out‐of‐plane direction in the AI phase below 75 K [see ZFC and FC curves in Figure 2(b)] may result from the characteristic behavior of Ising‐type 2D ferromagnetism. According to the Ising model, the thickness of the layered FGT is not a critical issue for magnetization of the layered FGT within the AI zone.

**Figure 5:**
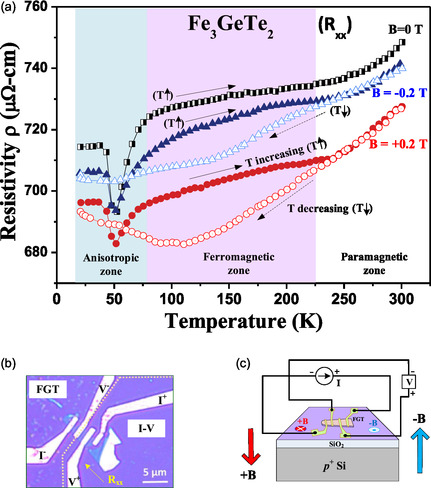
Temperature‐dependent longitudinal resistivity (R_xx_) with and without an applied magnetic field. a) Temperature‐dependent R_xx_ values obtained in the warming mode (T↑) under B = 0, B = −0.2, and B = +0.2 T conditions and in the cooling mode (T↓) under B = −0.2 and B = +0.2 T conditions. b) OM image of the multilayered FGT nanoflake on SiO_2_/Si used for magnetoresistance measurements. c) Illustration of the four‐point resistivity measurement with applied reversible magnetic fields of +B and −B.

The longitudinal in‐plane resistivity is also examined with and without applied magnetic fields to further confirm the transition temperatures between the AI, FM, and PM phases. **Figure** **5**(a) displays the longitudinal in‐plane resistivity (R_xx_) of multilayered FGT, applying reversible magnetic fields (B = −0.2 and +0.2 T) or no magnetic field (B = 0 T), using warming (T increasing, T↑) and cooling (T decreasing, T↓) modes between 20 and 300 K. The resistivity without a magnetic field was only measured with the T↑ mode, as shown in Figure 5(a), and the OM image of the multilayered FGT sample (thickness *t *≈ 45 nm) with the four‐point‐measurement arrangement (bar type) is shown in Figure 5(b). The multilayered FGT sample was exfoliated and transferred onto a SiO_2_/Si substrate, and Au/Ti contacts were patterned by electron‐beam evaporation. A representative scheme of the R_xx_ measurement with reversible magnetic fields (B = −0.2 and +0.2 T) is illustrated in Figure 5(c). It is noted that SiO_2_ and Si are diamagnetic insulator and semiconductor, which does not affect the magnetic and electrical behaviors of the FGT from low to room temperature. As shown in Figure 5(a), the warming mode (T↑) R_xx_ curves for B = 0, B = −0.2, and B = +0.2 T demonstrate a similar trend (shape) across the entire temperature range. The three R_xx_ curves show a metallic behavior as the temperature increases from 20 to 300 K while the temperature‐resistivity shift reveals different slopes in the AI, FM, and PM zones with different spin‐order phases. All the R_xx_ values in Figure 5(a) lie between 680 and 750 μΩ cm, verifying the metallic property of layered FGT, consistent with the DFT band‐structure and DOS calculations in Figure 2(d) and 3. In the AI zone below 75 K (light blue region), the R_xx_ curves for B = 0, −0.2, and +0.2 T in the T↑ mode show a resistivity drop (around 50 K) owing to an increase in the electron mobility caused by the well‐aligned spin electrons along the out‐of‐plane direction. The resistivity drops for the B = 0 T and B = −0.2 T curves are similar, ≈691 μΩ cm. This may be attributed to their similar spin‐moment directions. However, the B = +0.2 T condition presents the lowest R_xx_ drop value in Figure 5(a). The opposite‐spin electrons obtained by applying B = +0.2 T may possess higher mobility, resulting in the lowest R_xx_ value compared with those of the B = −0.2 and B = 0 T conditions. This can also be verified by the R_xx_ curves in the FM zone at 75–225 K (light red region), shown in Figure 5(a). Specifically, the R_xx_ curves for B = −0.2 and B = +0.2 T in the T↑ mode present negative magnetoresistances, and the R_xx_ value for B = +0.2 T with opposite spin exhibits the lowest resistivity compared with that for B = −0.2 T. The magnetic hysteresis window of R_xx_ for the T↑ mode in the FM region can also be determined based on the resistivity difference between the B = −0.2 and B = +0.2 T conditions in Figure 5(a). In contrast to the T↑ mode, the R_xx_ curves of the T↓ mode show distinct resistivity values in the FM and AI zones for both the B = −0.2 and B = +0.2 T conditions. However, in the PM zone at 225–300 K (white region), the R_xx_ values are matched and comparable for both B = −0.2 and *B* = +0.2 T. The random spin order of electrons in the PM phase loses the condensed electron spin. The phenomenon is distinction to the FM phase of FGT. Therefore, the R_xx_ values are matched in both the T↑ and T↓ modes under applied magnetic fields in the PM zone. Nevertheless, the cooling (T↓ and from PM to FM) and warming (T↑ and from FM to PM) modes of a magnet in the FM phase under applied magnetic fields (e.g., B = −0.2 and B = +0.2 T) should exhibit different spin order. Thus, the R_xx_ curves are separated and show distinct behavior as the temperature changes below the Curie temperature in Figure 5(a). The hysteresis curve of magneto‐resistivity with warming and cooling modes can also be observed in other oxide magnets like La_0.5_Ca_0.5_MnO_3_.^[^ ^47^ ^]^ Moreover, the resistivity drop of the R_xx_ curves in the T↓ mode was not found for the B = −0.2 and B = +0.2 T conditions (open symbols). For the FC condition (T↓ mode) from the PM to FM phase below 225 K, the spin electrons may be confined and condensed by external magnetic fields. Even if the temperature is lowered from the FM to AI phases below 75 K, the spin electrons may still be constrained by external magnetic fields, which hinders a significant change in the R_xx_ drop, as observed in Figure 5(a). Ultimately, the magnetoresistance effect makes multilayered FGT a potential candidate for applications in magnetic digital memory available for electronics or spintronics devices.

## 3 Conclusion

High‐quality layered single crystals of FGT were grown by CVT using I_2_ as the transport agent. XRD, HRTEM, and EDS results confirmed the hexagonal layered structure and 3:1:2 stoichiometric‐ratio content. Temperature‐dependent resistivity, Hall measurement, and theoretical band‐structure and DOS calculations revealed its n‐type metallic behavior, with Fe 3*d* electrons dominating the VBM and CBM band edges, thereby determining the magnetic, electrical, and optical properties. The temperature‐dependent μTR measurement of a ≈45 nm FGT nanoflake on a SiO_2_/Si substrate indicated that one A1 feature exists in the range of 20–300 K, for all the magnetic phases of the AI, FM, and PM zones. The A2 transition (energy above A1) is detected only below 75 K in the AI zone. The A3 transition (highest energy) is detected only at 20–220 K in the FM phase, disappearing in the PM zone at 225–300 K. According to the spin‐polarized band structure and total and partial DOS calculations, the A1, A2, and A3 μTR features may originate from the spin‐down states and the spin‐down band structure with Fe *d*–*d* transitions. The A1 feature is detectable independent of the spin ordering in the AI, FM, and PM phases. This may arise from the band‐edge transition of the spin‐down band structure at the Γ point. The A2 transition originates from the critical‐point transition with a parallel band slope in the Γ‐M direction. It occurred at the AI zone of low temperature with the spin moment of electrons largely along the out‐of‐plane directions. The A3 transition is found at the K point where the CB state becomes flatter to broaden and weaken the transition intensity in the PM region with random spin order. Temperature‐dependent magnetization, μRaman, XRD, and magnetoresistance measurements were implemented to verify the physical behaviors of the AI, FM, and PM phases. The transition temperatures between the AI and FM zone and between the FM and PM phases are 75 and 225 K, respectively, matching well with the temperature‐dependent μTR observations. In summary, the μTR technique was employed for the first time to characterize the optical transitions of the different magnetic phases in the multilayered FGT metal. The results were validated and the technique can be further utilized to characterize band structures and optical transitions of the other 2D layered magnets.

## 4 Experimental Section

4.1

4.1.1

##### Crystal Growth of Bulk Single Crystals

Single crystals of FGT were grown by CVT using I_2_ as the transport agent. The high‐purity powdered materials (Fe: 99.999%, Ge: 99.99%, and Te: 99.999%) with stoichiometric composition (Fe:Ge:Te ratio is 3:1:2, with 10 g of powder in total) were first combined with an appropriate amount of I_2_ (12 mg cm^−3^) and placed in a quartz ampoule (20 cm length and 2 cm inner diameter). The quartz ampoule was directly cooled with liquid nitrogen and then sealed in a vacuum environment at ≈10^−6^ Torr. The ampoule was then heated at 750 °C for two days in a three‐zone furnace to synthesize the source material. Next, the furnace temperature was adjusted to create a temperature gradient of 750 °C (source zone) à 650 °C (growth zone) for crystal growth. The I_2_ acted as a transport agent and facilitated the vapor transport of FGT from the source zone to the growth zone, where nucleation and crystal growth occur. The growth process lasted for 240 h, yielding large, gray, shiny layered crystals with areas of up to ≈0.8 cm^2^ and thicknesses of up to 300 μm. The FGT crystals had a layered structure with weak vdW interaction between the layers, which enables the mechanical exfoliation and transfer of multilayered FGT nanoflakes onto SiO_2_/Si substrates using adhesive tapes of different stickiness. Finally, FGT nanoflakes were prepared with different thicknesses (≈40–100 nm) and transferred on SiO_2_/Si for μTR, μRaman, and magnetoresistance measurements.

##### Structural Study of FGT from Electronic and Optical Characterization

Structural properties of FGT were initially characterized by powered XRD using a Bruker D2 PHASER XE‐T instrument with a Cu Kα (*λ* = 0.15406 nm) X‐ray source. Samples were finely ground, and XRD measurements were conducted over a 2θ range of 20°–80°. In‐plane lattice constants and stoichiometry were further determined by HRTEM using a Tecnai F20 G2 TEM instrument equipped with an EDS detector. The HRTEM images and SAED patterns were obtained from thin, exfoliated FGT mounted on Cu mesh holders. XPS was performed using a ULVAC‐PHI spectrometer to quantify the elemental composition and identify the energy states of the elements. μRaman spectroscopy was employed using a ProTrusTech Ramaker spectrometer equipped with a 532 nm solid‐state laser, an Olympus 50× objective, and an Andor CCD spectrometer with a 1200 grooves/mm grating. A pair of dichroic sheet polarizers, designed for the visible‐to‐infrared range, was mounted on a rotatable holder and used for angle‐dependent polarized measurements.

##### μTR Measurements

μTR spectroscopy was performed using a white light derived from a 150 W tungsten halogen lamp, which was dispersed using a 0.2 m Photonics International monochromator equipped with a grating (1200 grooves/mm) to provide monochromatic light. The SiO_2_/Si substrate decorated with multilayered FGT (substrate size ≈0.8 × 0.8 × 0.01 cm) was firmly attached to an Au‐covered quartz plate. The monochromatic light source was coupled onto the few‐layer or multilayer sample using a silica fiber, and it passed through a light‐guiding microscope (LGM) equipped with an Olympus objective lens (50×, working distance ≈8 mm). This served as the interconnection coupled medium between the few‐layer sample and the incident and reflected light.^[^ ^36^ ^]^ The reflected light from the layered sample was collected by the LGM and then coupled to an EG&G HUV200B Si detector using an additional silica fiber. Optical alignment was accomplished by the XYZ adjustment of the nanoflake sample using a charge‐couple device (CCD) imaging camera in the LGM. A 4 Hz heating current (≈0.5 A) was periodically supplied to the Au heater for thermal modulation of the sample's lattice constant and band edge. Phase‐sensitive detection was achieved using an NF 5610B lock‐in amplifier. A Janis open‐circled liquid‐helium cryostat equipped with a Lakeshore 335 thermometer controller was used to facilitate temperature‐dependent measurements from low to room temperature.

##### Computational Methods

Spin‐polarized DFT calculations were performed to optimize the geometries using Quantum Espresso.^[^ ^44^, ^48^ ^]^ Projector augmented wave potentials were utilized for the description of the electronic structure, and the exchange–correlation was modeled using the generalized gradient approximation of Perdew–Burke–Ernzerhof with the D3 dispersion correction scheme.^[^ ^49^, ^50^ ^]^ The cutoffs of 90 and 1080 Ry were implemented for the plane wave expansion and charge density, respectively. The optimized structures were obtained by relaxing all atomic positions using the Broyden−Fletcher−Goldfarb−Shanno quasi‐Newton algorithm until all forces were smaller than 1 × 10^−3^ Ry/Bohr, and the energy convergence of two consecutive steps was 1 × 10^−4^ Ry. An 8 × 8 × 2 Monkhorst–Pack grid was used for Brillouin zone integration, and DOS calculations were performed with a double *k*‐point grid using the tetrahedron method to sample the entire first Brillouin zone, ensuring high accuracy.

##### Magnetic Field‐Dependent Resistivity Measurement of Multilayered FGT

Multilayer FGT with a thickness of ≈45 nm was mechanically exfoliated from the bulk using tape. Polydimethylsiloxane (PDMS) was used as a medium to transfer and deposit these multilayered materials onto a SiO_2_/Si substrate (size ≈0.8 × 0.8 × 0.01 cm^3^), which was then patterned with Au/Ti electrodes in a four‐point configuration using an electron‐beam evaporator. Precise alignment of the nanoflake during transfer was achieved using a microscope stage with three axial micromanipulators. AFM was employed to measure the height profile of the material. The FGT decorated SiO_2_/Si substrate was then mounted on a copper holder with an outside Al cover for thermal shielding. The vacuum was evacuated to ≈10^−4^ Torr. A closed‐cycle He compressor system equipped with a thermometer controller facilitated the temperature‐dependent measurement between 20 and 300 K (including warming and cooling modes). Current‐voltage measurements were implemented using a Keithley 6220 programmable current source and a Keithley 2182 nanovoltmeter. A pair of permanent magnets was attached on the outside of the Al cover to provide reversible magnetic fields of  ±0.2 T by rotating the Al outer cover 0° or 180°.

## Conflict of Interest

The authors declare no conflict of interest.

## Author Contributions

**Ching‐Hwa Ho**: conceived the idea and supervised the optical, magnetic, and structural characterization. **Zi‐Ying Chen** and **Ching‐Hwa Ho**: grew the samples. **Ching‐Hwa Ho**: is responsible for funding acquisition. **Yen‐Chang Su** and **Zi‐Ying Chen**: maintained and performed microthermoreflectance experiments. **Yu‐Hung Peng**: performed HRTEM and magnetic field‐dependent resistivity measurements. **Ching‐Hwa Ho**: conducted theoretical calculations and analyzed the theoretical and experimental data. **Ching‐Hwa Ho**: wrote the manuscript.

## Supporting information

Supplementary Material

## Data Availability

The data that support the findings of this study are available from the corresponding author upon reasonable request.
