# Supplementary material for: Microthermoreflectance Characterization of the Band‐Structure Transformations Observed During the Magnetic‐Ordering Transitions of Multilayered 2D Fe3GeTe2 Ferromagnetic Metals
Source: Small Sci. 2025 Aug 13;5(11):2500293. doi: 10.1002/smsc.202500293 (PMC12622467; doi:10.1002/smsc.202500293)
Supplement: Supplementary file 1 — Supplementary Material [file SMSC-5-2500293-s001.pdf]

Supplementary Information of

Micro-thermoreflectance Characterization of the Band-structure Transformations Observed during the Magnetic-ordering Transitions of Multilayered 2D Fe_3GeTe_2 Ferromagnetic Metals

Ching-Hwa Ho,[a,b,*] Yen-Chang Su,[a] Yu-Hung Peng,[a] and Zi-Ying Chen[a]

[a] *Graduate Institute of Applied Science and Technology, National Taiwan University of Science and Technology, Taipei 106, Taiwan*

[b] *Taiwan Consortium of Emergent Crystalline Materials (TCECM), National Science and Technology Council, Taipei, 106, Taiwan*

[*]Corresponding author, E-mail address: chho@mail.ntust.edu.tw

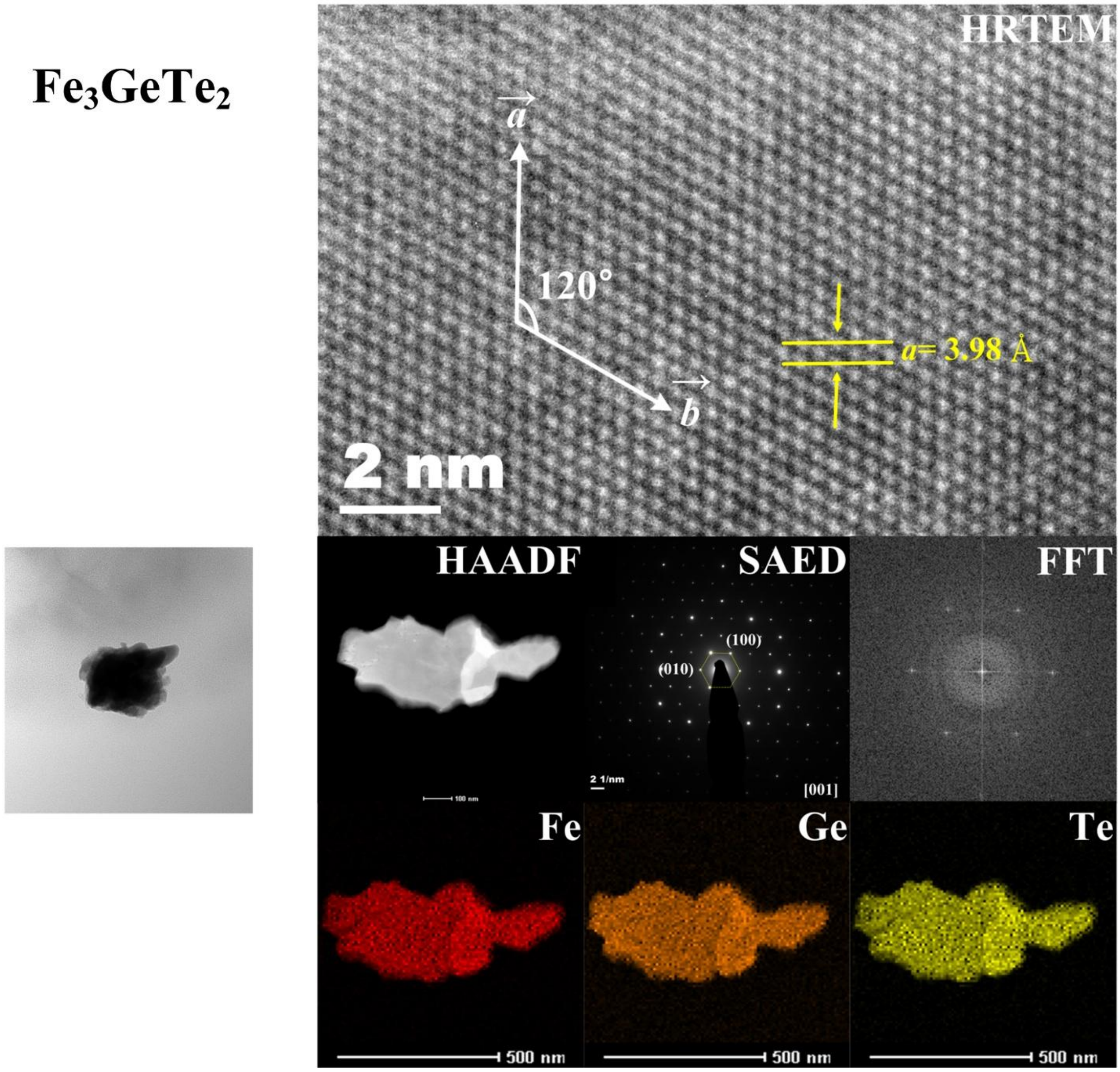

Figure S1. TEM, SAED, and EDS results for FGT nanoflakes. The left image shows the exfoliated FGT nanoflake, and the upper-right photo reveals the HRTEM result of the basal plane with an in-plane lattice constant of a = 3.98 Å and hexagonal angle of 120°. The HAADF-STEM result, SAED pattern, and fast Fourier transform image are displayed in the middle of the right part. The lower portion of the right part shows the EDS mapping of each element (Fe, Ge, and Te). The measured elemental composition matches well with the nominal composition of FGT.

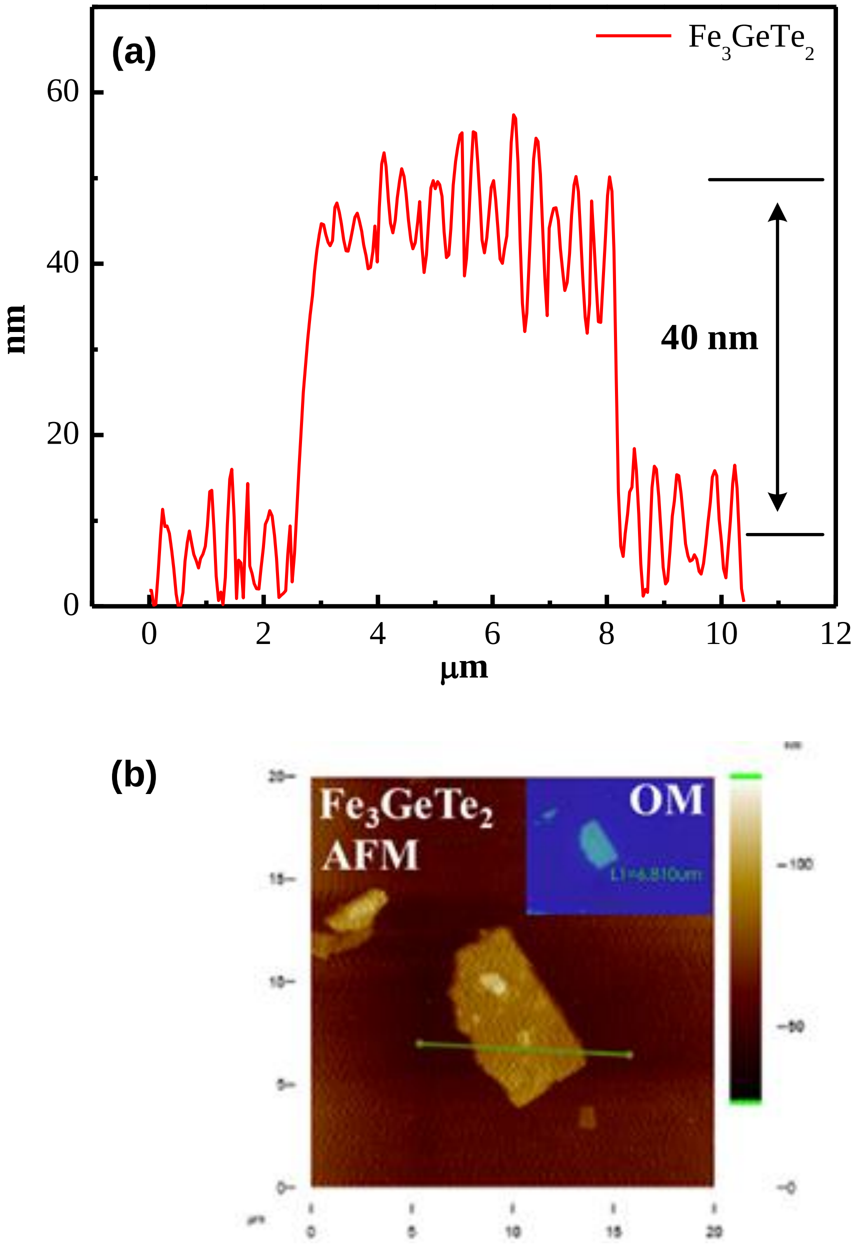

Figure S2. AFM and OM of a multilayered FGT nanoflake on SiO_2/Si. (a) Thickness profile. (b) AFM image of the area used for measuring the thickness profile. The inset shows the OM image observed in the μTR LGM system.

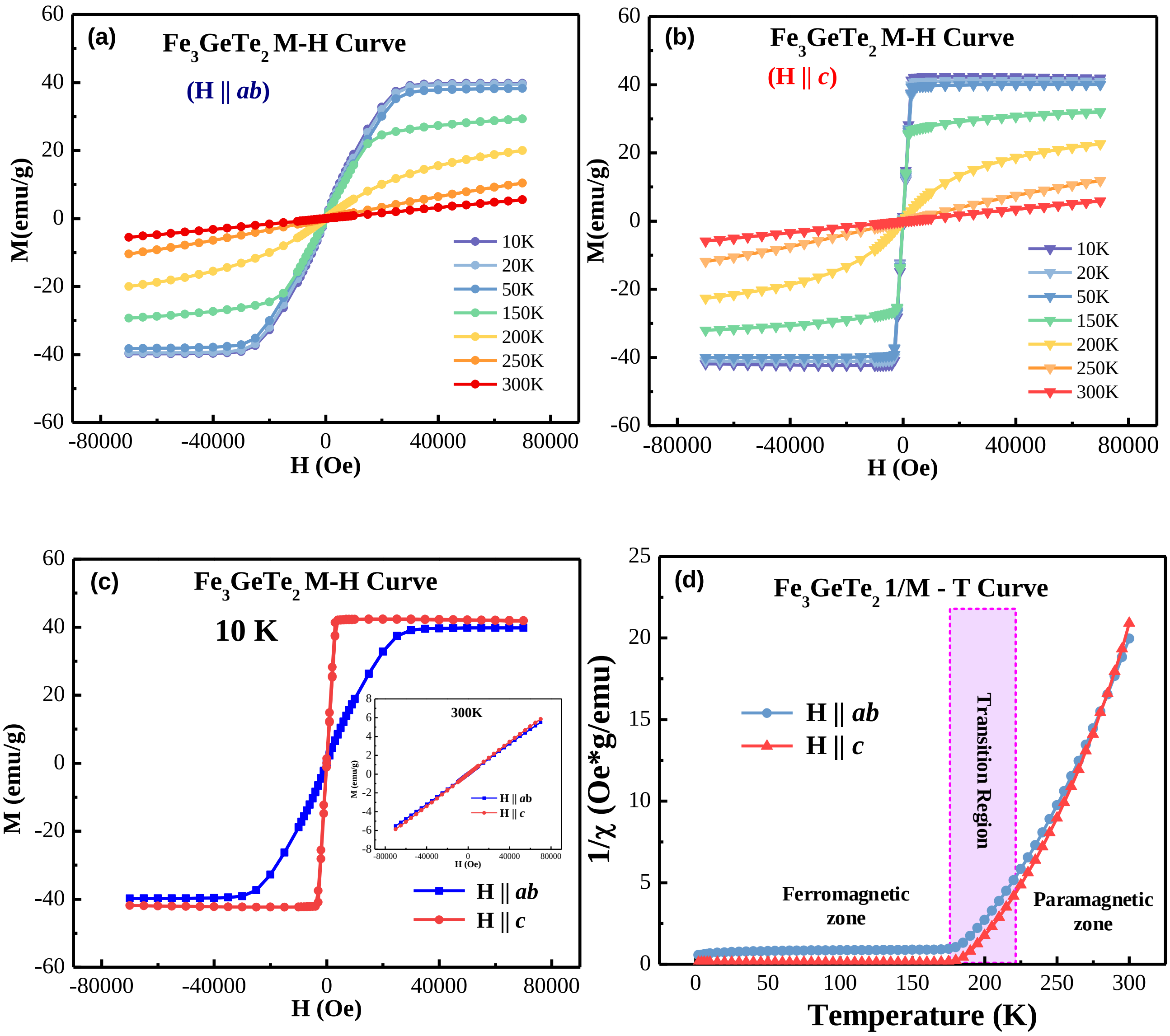

Figure S3. In-plane and out-of-plane magnetization curves for FGT. (a) In-plane M-H curves between −70000 to 70000 Oe with the H||*ab* configuration between 10 and 300 K. (b) Out-of-plane M-H curves between −70000 to 70000 Oe with the H||*c* configuration between 10 and 300 K. The M-H curves become linear at 250 and 300 K in (a) and (b), implying that the FGT sample is in the PM phase. (c) Comparison of the in-plane (H||*ab*) and out-of-plane (H||*c*) magnetization curves at 10 K in the AI zone. The inset shows the magnetization curves at 300 K in the PM zone. (d) Temperature-dependent magnetization 1/M versus T curves for the in-plane (H||*ab*) and out-of-plane (H||*c*) conditions. The light red area marks the transition region between the PM and FM phases.

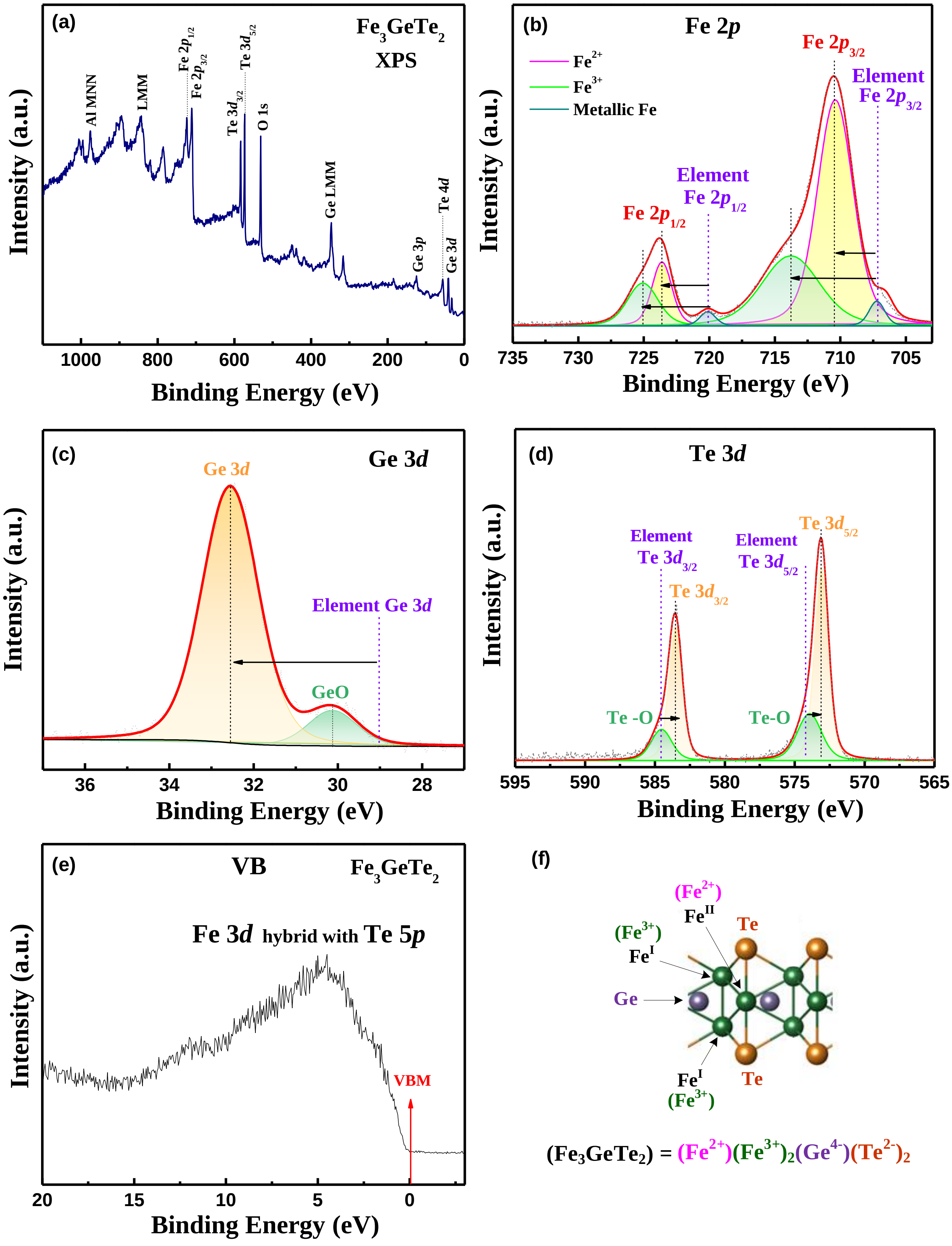

Figure S4. XPS results for FGT. (a) Full XPS spectrum of FGT with characteristic peaks. (b) Fe 2*p* states ($2p_{1/2}$ and $2p_{3/2}$) of Fe^{II} (Fe^{2+}), Fe^{I} (Fe^{3+}), and pure Fe. The atomic positions of Fe^{I}, Fe^{II}, Ge, and Te in FGT are shown in (f). The Ge 3*d*, Te 3*d*, and VB states of FGT are shown in (c), (d), and (e),

respectively. (f) Side view of the atomic arrangement for Fe^{I} (Fe^{3+}), Fe^{II} (Fe^{2+}), Ge, and Te in FGT. The matched valence of FGT can be recognized as $Fe_3GeTe_2 = (Fe^{2+})(Fe^{3+})_2(Ge^{4-})(Te^{2-})_2$. From (b) (c) and (d), the Fe 2*p* states ($2p_{1/2}$ and $2p_{3/2}$) and Ge 3*d* states show energy increase from pure element shifted to the FGT compound status while the Te 3*d* states ($3d_{3/2}$ and $2d_{5/2}$) present energy decrease from pure element to the compound status for identification of the cation and anion properties of the layered Fe_3GeTe_2. From (e) the VB states near the VBM are from Fe 3*d* hybridization with Te 5*p* recognized from the band-structure and DOS calculations in Figures 2(d) and 3.

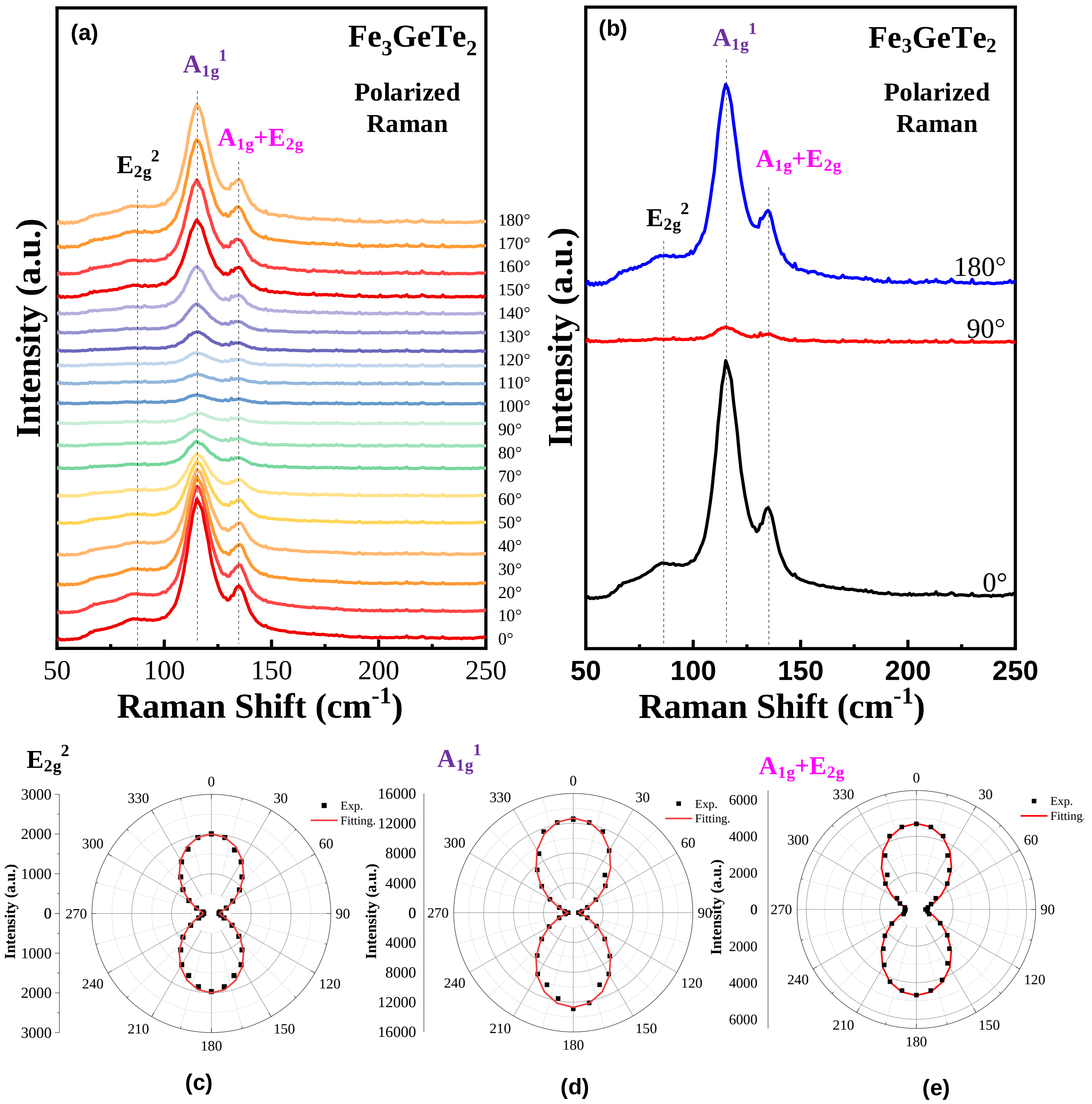

Figure S5. Angular dependence of polarized μRaman results for layered FGT. (a) Polarized Raman results obtained between 0 and 180°. (b) Polarized Raman spectra of 0°, 90°, and 180°. Polar plots of the (c) E_{2g}^2, (d) A_{1g}^1, and (e) A_{1g}+E_{2g} modes, which are analyzed by $I(\theta) = I_0 + I_P \times cos^2(\theta - \theta_m)$, where $\theta = 0°$ is the direction of a-axis. The red lines show the fitted results, and the obtained fitting parameters are I_0 = 30, 200, and 500, I_p = 300, 11000, and 6500, and θ_m = 0.12°, 0.1°, and 0.1° for the E_{2g}^2, A_{1g}^1, and A_{1g}+E_{2g} modes, respectively. The out-of-plane vibration mode of A_{1g}^1 exhibits the highest polarized rejection ratio among the three Raman modes.
